# High-Resolution Single-Shot Fast Spin-Echo MR Imaging with Deep Learning Reconstruction Algorithm Can Improve Repeatability and Reproducibility of Follicle Counting

**DOI:** 10.3390/jcm12093234

**Published:** 2023-04-30

**Authors:** Renjie Yang, Yujie Zou, Weiyin (Vivian) Liu, Changsheng Liu, Zhi Wen, Liang Li, Chenyu Sun, Min Hu, Yunfei Zha

**Affiliations:** 1 Department of Radiology, Renmin Hospital of Wuhan University, Wuhan 430060, China; 2 Reproductive Medicine Center, Renmin Hospital of Wuhan University, Wuhan 430060, China; 3 MR Research, GE Healthcare, Beijing 100080, China; 4 First School of Clinical Medicine of Wuhan University, Wuhan 430060, China; 5 Department of Obstetrics, Renmin Hospital of Wuhan University, Wuhan 430060, China

**Keywords:** single-shot fast spin-echo, deep learning reconstruction, fast spin-echo, polycystic ovary syndrome, follicle count, follicle number per ovary

## Abstract

Objective: To investigate the diagnostic performance of high-resolution single-shot fast spin-echo (SSFSE) imaging with deep learning (DL) reconstruction algorithm on follicle counting and compare it with original SSFSE images and conventional fast spin-echo (FSE) images. Methods: This study included 20 participants (40 ovaries) with clinically confirmed polycystic ovary syndrome (PCOS) who underwent high-resolution ovary MRI, including three-plane T2-weighted FSE sequences and slice-matched T2-weighted SSFSE sequences. A DL reconstruction algorithm was applied to the SSFSE sequences to generate SSFSE-DL images, and the original SSFSE images were also saved. Subjective evaluations such as the blurring artifacts, subjective noise, and clarity of the follicles on the SSFSE-DL, SSFSE, and conventional FSE images were independently conducted by two observers. Intra-class correlation coefficients and Bland–Altman plots were used to present the repeatability and reproducibility of the follicle number per ovary (FNPO) based on the three types of images. Results: SSFSE-DL images showed less blurring artifact, subjective noise, and better clarity of the follicles than SSFSE and FSE (*p* < 0.05). For the repeatability of the FNPO, SSFSE-DL showed the highest intra-observer (ICC = 0.930; 95% CI: 0.878–0.962) and inter-observer (ICC = 0.914; 95% CI: 0.843–0.953) agreements. The inter-observer 95% limits of agreement (LOA) for SSFSE-DL, SSFSE, and FSE ranged from −3.7 to 4.5, −4.4 to 7.0, and −7.1 to 7.6, respectively. The intra-observer 95% LOA for SSFSE-DL, SSFSE, and FSE ranged from −3.5 to 4.0, −5.1 to 6.1, and −5.7 to 4.2, respectively. The absolute values of intra-observer and inter-observer differences for SSFSE-DL were significantly lower than those for SSFSE and FSE (*p* < 0.05). Conclusions: Compared with the original SSFSE images and the conventional FSE images, high-resolution SSFSE images with DL reconstruction algorithm can better display follicles, thus improving FNPO assessment.

## 1. Introduction

Polycystic ovary syndrome (PCOS) is the most common endocrine disorder in women of reproductive age, affecting 6–15% of this population [1]. The diagnosis of PCOS needs to meet the following two of three criteria: clinically or biochemically confirmed hyperandrogenism, irregular menstrual cycles, and polycystic ovary morphology (PCOM) [2]. Therefore, the determination of PCOM is of great value when the diagnosis of PCOS is uncertain according to the clinical manifestations and laboratory findings. The main markers of PCOM are an increase in ovarian volume (OV) and an excess of follicles, with the latter being more diagnostic than the former [3].

According to the revised criteria from the 2003 Rotterdam Consensus Workshop, PCOM was defined as 12 or more follicles at the size of 2–9 mm per ovary and/or OV greater than 10 cm^3^ in adults by using transvaginal ultrasonography (TVUS) [2]. However, the Androgen Excess and Polycystic Ovary Syndrome Society Task Force recommended increasing the threshold value of follicle number per ovary (FNPO) to 25 for PCOM determination when using advanced ultrasound scanners with improved resolution [3]. The latest international evidence-based guideline has recommended that the threshold value of FNPO should be set at 20 follicles when using newer technology (i.e., transducer frequency ≥ 8 MHz) [4]. The used thresholds of follicle counting using TVUS in adult women varied [3,4,5,6], not to mention age-specific diagnostic criteria for adolescents, in whom TVUS is generally contraindicated. However, TVUS still remains the most widely used imaging measure due to its convenience, safety, and low costs [3,4,7]. The accuracy of follicle count estimation is also low due to its inherent disadvantages, such as operator dependence and limited field-of-view. Furthermore, the poor resolution of the transabdominal pelvic ultrasound in adolescents and women with no sexual history makes it more difficult to perform reliable follicle counting.

Not limited by the observation field-of-view, patient size, or sexual history, magnetic resonance imaging (MRI) is increasingly used as an alternative to TVUS in the characterization of the ovarian morphology in both adult women and adolescents with suspected PCOS [5,7,8]. It has been confirmed that two-dimensional (2D) MRI sequences are more effective than TVUS for detecting small follicles and thus have been recommended as a better method for follicle counting [9,10]. Fast spin-echo (FSE) based T2-weighted imaging (T2WI) is currently the mainstay of ovary MRI for the evaluation of polycystic ovary [5,11,12]. Due to the lack of standard imaging protocols, the repeatability of follicle counting is still controversial. To obtain a reliable assessment of follicle counting, MR images with high spatial resolution and signal-to-noise ratio (SNR) could better delineate the follicles with various sizes.

FSE imaging is relatively easy to achieve high SNR and spatial resolution at the expense of acquisition time, which may lead to increased motion artifacts. Single-shot FSE (SSFSE), as a fast imaging sequence, is not sensitive to motion, but it is not a preferred sequence for the ovary MRI due to its blurring and relatively low SNR, especially when high-resolution imaging is pursued [13,14]. Recently, deep learning (DL) reconstruction algorithm has been widely used in medical imaging and shows great potential in improving SNR for high-resolution imaging without increasing scanning time [15,16]. As far as we know, no study has investigated the diagnostic efficacy of the DL-reconstructed SSFSE (SSFSE-DL) for follicle counting. In this study, we aimed to establish an MR imaging protocol with higher reproducibility of follicle number via comparisons of the repeatability of follicle counts based on original SSFSE, SSFSE-DL, and conventional FSE images.

## 2. Materials and Methods

### 2.1. Subjects

This prospective single-center study was approved by the Clinical Ethics Committee of Renmin Hospital of Wuhan University (WDRY2021-K028) and followed the principles of the Declaration of Helsinki. We performed a prior analysis using G. power to determine the required sample size for the comparison of subjective scores of SSFSE with DL or without DL and FSE images. The analysis indicated that a minimum of 34 follicle counts were needed to ensure a test efficiency of ≥0.8. We prospectively recruited a total of 22 clinically confirmed PCOS cases, including 6 adolescent girls and 16 adult women, from the departments of gynecology, endocrinology, reproductive medicine, and pediatrics at Renmin Hospital of Wuhan University between June 2022 and November 2022. The diagnosis of PCOS in adult women met at least two of the following signs: clinically or biochemically confirmed hyperandrogenism, irregular menstrual cycles, and PCOM [2]. For adolescents, both the criteria of hyperandrogenism and irregular cycles are needed. Informed consent was obtained from all participants aged ≥ 18 years. For participants under the age of 18, consent of the parents and participants were both required. Patients aged 14–35 years with clinically confirmed PCOS would be considered to be recruited. Concurrently, patients who (a) were pregnant, (b) had ever received ovarian surgery, or (c) had contraindications for MRI, or (d) could not tolerate the MRI examination would be excluded.

### 2.2. Magnetic Resonance Imaging

All participants underwent ovary MR imaging on a 3-T MR imaging unit (SIGNA Architect; GE Medical Systems, Milwaukee, WI, USA) with a 30-channel phased-array coil. The feet-first supine position was adopted for all the participants during the MRI examination. Sedation or injection of contrast agents is not used for good patient compliance during the examination. All MRI protocols consisted of three-plane (axial, coronal, sagittal) T2-weighted FSE sequences (TR/TE = 4214–5478 ms/104 ms; receiver bandwidth = ±50 kHz; slice thickness/spacing = 3 mm/0 mm; ETL = 22; number of excitations = 2; FOV = 18 cm; acquisition matrix (frequency × phase) = 352 × 352 and total scan time = 6 min 30 s–7 min 15 s) and matched three-plane T2-weighted SSFSE sequences (TR/TE = 1700 ms/84 ms; receiver bandwidth = ±62.5 kHz; slice thickness/spacing = 3 mm/0 mm; number of excitations = 1; FOV = 18 cm; acquisition matrix (frequency × phase) = 352 × 352 and total scan time = 1 min 30 s–1 min 54 s). A commercially available DL reconstruction algorithm (AIR^TM^ Recon DL, GE Healthcare) was used in the SSFSE sequences (abbreviated as SSFSE-DL), and the original images (abbreviated as SSFSE) were also saved. There were finally three image types, including SSFSE, SSFSE-DL, and FSE, for each participant.

### 2.3. Qualitative Analysis

Three types of axial T2-weighted images for each participant were evaluated for the display performance of the follicles. A radiologist with 5 years of diagnostic experience in pelvic MRI randomized the 60 image sets before analyses. Two other radiologists who were blinded to the imaging and clinical information assessed bilateral ovaries on each participant independently and subjectively. The evaluation items were as follows: blurring artifacts on a 3-point scale (3 = almost no blurring artifacts, 2 = mild blurring artifacts, and 1 = severe blurring artifacts); subjective noise on a 3-point scale (3 = almost no noise, 2 = mild noise, 1 = severe noise); clarity of the follicles on a 5-point scale (5 = excellent, 4 = good, 3 = fair, 2 = poor, 1 = uninterpretable).

### 2.4. Assessment of Follicle Number Per Ovary (FNPO)

The FNPO was determined by counting the number of follicles measuring ≥ 1 mm long axis based on each image type. To avoid a repeat count of the follicles in different slices, the follicles were counted on the axial images, and the corresponding sagittal and coronal images would be referred to at the same time. The assessments of FNPO for both ovaries on each participant were performed twice by observer 1 (L.L., a board-certified radiologist with more than 10 years of experience in pelvic MRI) and once by observer 2 (Z.W., a third-year radiology resident with 6 months of training in pelvic MRI). To avoid any potential recall bias, there was an interval of 4 weeks between two assessments by observer 1.

### 2.5. Statistical Analysis

The subjective scoring data were reported as mean ± standard deviation (SD). Qualitative results were compared between SSFSE-DL and SSFSE and between SSFSE-DL and FSE by using Wilcoxon signed-rank tests. Cohen’s weighted kappa was used to assess inter-observer agreement. Kappa values of 0.00–0.20, 0.20–0.40, 0.40–0.60, 0.60–0.80, and 0.80–1.00 indicated poor, fair, moderate, good, and excellent agreement, respectively [17]. Confidence intervals (CIs) for the kappa values were calculated.

Intra-class correlation coefficients (ICCs) were calculated to show the intra-observer and inter-observer agreements for FNPO assessment, including FNPO assessment by reviewer 1 for the first and second session and reviewer 1 and reviewer 2 for the first session. The agreement levels were as follows: ICC greater than 0.80, almost perfect agreement; 0.61–0.80, substantial agreement; 0.41–0.60, moderate agreement; 0.21–0.40, fair agreement; 0.0–0.2, slight agreement; and less than 0.0, poor agreement. Intra-observer and inter-observer variabilities were assessed by Bland–Altman plots [18]. The mean difference and 95% limits of agreement (LOA), which are defined as the mean difference ± 1.96 SDs, were calculated. To further assess the significance of the intra-observer and inter-observer differences between SSFSE-DL and SSFSE and between SSFSE-DL and FSE, the absolute values of the intra-observer and inter-observer differences were compared by using a paired *t* test. A paired *t* test was also used to compare the repeatability and reproducibility within each image group.

Statistical analyses were performed using MedCalc Software (v. 11.6, Nariakerke, Belgium). *p* < 0.05 was considered statistically significant.

## 3. Results

### 3.1. Study Participants

Among the recruited 22 participants, one adult woman was excluded because of the surgery of the right ovary for ovarian teratoma, and one adolescent girl was excluded due to her intolerance during the MRI examination. Finally, this study included 20 Chinese women (5 adolescent girls and 15 adult women) with a mean age of 22.5 ± 4.7 years (range: 15–31 years), mean gynecological age of 10.5 ± 4.9 years (range: 3–20 years), and mean body mass index (BMI) of 24.2 ± 3.6 kg/m^2^ (range: 16.3–32.2 years). Four adolescent girls admitted no sexual history, and 1 adolescent girl and all 15 adult women had a sexual history. A total of 40 ovaries were included for further evaluation.

### 3.2. Qualitative Image Analysis

**Figure 1:**
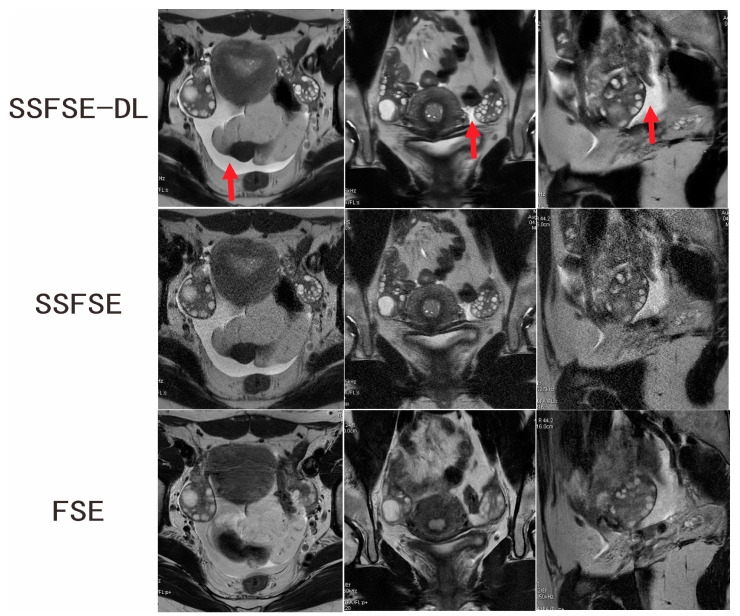
Ovarian MRI in a 16-year-old adolescent girl with confirmed PCOS. SSFSE-DL images (upper row) show the lowest noise, blurring artifacts, and the bilateral enlarged ovaries with a dominant follicle and many small follicles. The pelvic fluid collection (arrow) is easily detected on the SSFSE-DL images. Noise is prominent on the SSFSE images (middle row). The FSE images (lower row) mainly showed blurring artifacts caused by respiratory and bowel motility.

**Figure 2:**
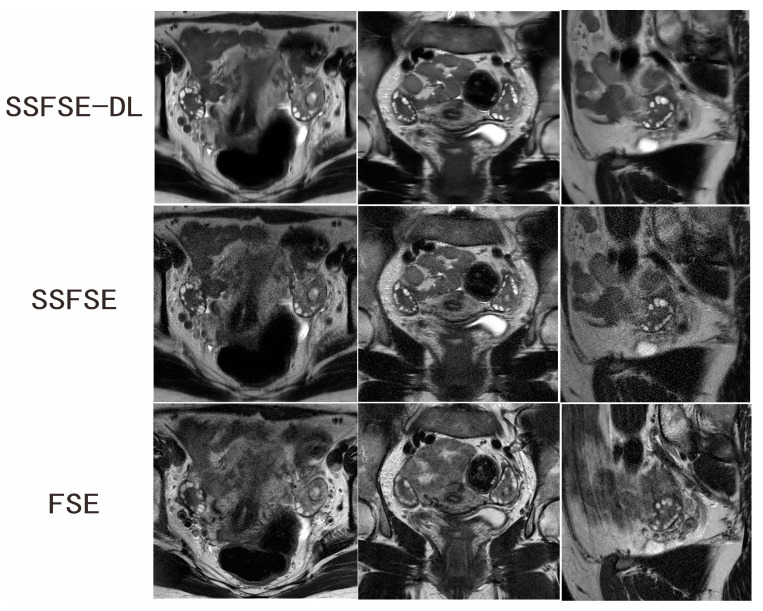
Ovarian MRI in a 28-year-old woman with confirmed PCOS. SSFSE-DL images (upper row) show the least noise and blurring artifacts. The bilateral enlarged ovaries with many small peripheral follicles are clearly delineated on the SSFSE-DL images. The display of the follicles is impaired by the noise on the SSFSE images (middle row) and by the blurring artifacts in the FSE images (lower row).

Two independent observers conducted the subjective assessment on a total of 40 ovaries in 60 images. Table 1 summarizes the comparison results of the qualitative image analyses between SSFSE-DL and SSFSE as well as between SSFSE-DL and FSE for each observer. Each subjective evaluation item was presented as mean ± SD. Significantly better scores of the blurring artifacts, subjective noise, and clarity of the follicles rated by observers were found between SSFSE-DL and SSFSE (*p* < 0.05) as well as FSE (*p* < 0.05). The use of SSFSE helped to reduce the blurring artifacts compared to the FSE sequences, and the application of DL reconstruction helped to reduce the noise of the high-resolution SSFSE images. Two typical cases are shown in Figure 1 and Figure 2. The inter-observer agreements of the three subjective aspects were good for SSFSE-DL (Kappa = 0.671–0.789), moderate to good for SSFSE (Kappa = 0.580–0.636), and FSE (Kappa = 0.474–0.664).

### 3.3. Repeatability and Reproducibility of FNPO Assessment

Table 2 presents the mean and range of FNPO based on each image type across all 40 ovaries according to each observer. The FNPO assessments for all 40 ovaries were performed twice by observer 1 and once by observer 2. For two assessment sessions conducted by observer 1 and for one session performed by observer 2, a range of 12 to 38 and 14 to 35 follicles on SSFSE-DL images, 12 to 32 and 14 to 31 follicles on SSFSE images, 11 to 32 and 12 to 34 follicles on FSE images were, respectively, reported. Table 3 summarizes the ICCs for the FNPO assessments according to each image type. SSFSE-DL showed the best intra-observer agreement as a higher ICC of 0.930 compared to SSFSE (0.825) and FSE (0.839). In addition, SSFSE-DL showed the best inter-observer agreement with a higher ICC of 0.914 compared to SSFSE (0.798) and FSE (0.669).

**Figure 3:**
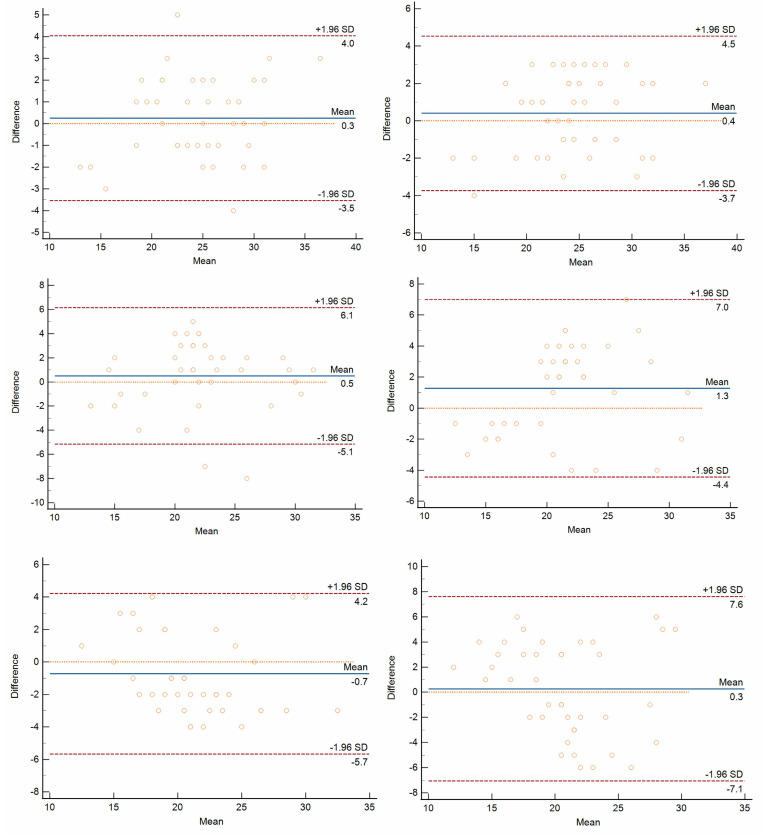
Bland–Altman plots show intra-observer (left column) and inter-observer (right column) variability of FNPO assessments based on SSFSE-DL (upper row), SSFSE (middle row) and FSE (lower row) images. The *x*-axis represents the mean of two measurements, and the *y*-axis represents the differences between the two measurements. The solid lines (blue) indicate the mean differences between all measurements. The upper dashed lines (red) indicate a mean difference of + 1.96 SD, and the lower dashed lines (red) indicate a mean difference of −1.96 SD. The limits of agreement (mean difference ± 1.96 SDs) are expected that the limits include 95% of the differences between the two measurements.

Bland–Altman plots in the representation of the intra-observer and inter-observer variability of FNPO assessments for each image type are presented in Figure 3. The mean differences of intra-observer were 0.3, 0.5, and −0.7 for SSFSE-DL, SSFSE, and FSE, respectively, and the mean differences for inter-observer were 0.4, 1.3, and 0.3 for SSFSE-DL, SSFSE and FSE, respectively. The 95% LOA of inter-observer for SSFSE-DL, SSFSE, and FSE ranged from −3.7 to 4.5, −4.4 to 7.0, and −7.1 to 7.6, respectively, and the 95% LOA of intra-observer for those ranged from −3.5 to 4.0, −5.1 to 6.1, and −5.7 to 4.2, respectively. SSFSE-DL showed the narrowest inter-observer and intra-observer 95% LOA compared to SSFSE and FSE. The absolute values of intra-observer and inter-observer differences were significantly lower for SSFSE-DL than for SSFSE and FSE (*p* < 0.05). SSFSE-DL showed significantly reduced intra-observer and inter-observer variability for the FNPO assessments. In addition, there were no significant differences between the absolute values of intra-observer and inter-observer differences neither for SSFSE-DL (*p* = 0.071) nor for SSFSE (*p* = 0.143). However, for FSE, the absolute values of intra-observer differences were significantly lower than those of inter-observer (*p* = 0.001) (Table 4).

## 4. Discussion

To the best of our knowledge, this is the first study evaluating the diagnostic performance of DL-reconstructed SSFSE on the antral follicle counts up to the present. Our data demonstrated that SSFSE-DL significantly improved image quality (i.e., reduced blurring artifacts and image noise). The counts of the follicles assessed by SSFSE-DL had the best intra-observer repeatability and inter-observer reproducibility for the FNPO assessment.

TVUS is currently the most widely used imaging measure to identify PCOM [19], and thus, the criteria for PCOM determination have been largely based on TVUS. Additionally, with the advancement of ultrasound scanners, the threshold value for the identification of PCOM has increased from 12 to 20 [2,3,4], highlighting the substantial impact of the resolution of TVUS on the accuracy of follicle counting. Nevertheless, when compared to 2D MRI, the follicle number is still likely to be underestimated. Wang et al. [10] compared 2D TVUS and 2D MRI for the estimation of follicle count in 84 adult women with infertility. They reported that the follicle counts at 1–9 mm and 1–3 mm assessed by 2D TVUS was about 8 and 13 follicles smaller than those evaluated by 2D MRI, respectively. Nylander et al. [20] compared 2D TVUS, 3D TVUS, and 2D MRI for the estimation of follicle count on 66 overweight women with PCOS and reported that the follicle count assessed by 2D TVUS was 18% and 16% smaller than those evaluated by 3D TVUS and 2D MRI, respectively. In a study conducted by Leonhardt et al. [9], the estimation of follicle count using 3D TVUS and 2D MRI in 99 women aged 21–37 years showed that the total follicle count by 2D MRI was approximately 14 follicles higher than that by 3D TVUS, in particular, the 1–3 mm follicle count by 2D MRI was 22 follicles more than that by 3D TVUS. In view of these above-mentioned findings, 2D MRI is an effective alternative to TVUS for detecting a greater number of small follicles. However, the inter-observer agreement for the small follicles by 2D MRI was found to be poor to moderate [9,10]. The spatial resolution of the 2D MR images is a key factor that affects the repeatability of the follicle count assessment.

To achieve high-resolution imaging of the ovary, an identical acquisition voxel of 0.5 × 0.5 × 3 mm^3^ was set for both SSFSE and FSE T2WI sequences in our study. The noise was well resolved in the FSE images at the expense of an acquisition time of approximately 2 min for each sequence. Severe image noise appeared in the SSFSE images on account of the excessively small voxel, let alone its inherently low SNR. The reason is that there is a trade-off between resolution and SNR when using the conventional inverse Fourier transform (iFT) reconstruction method [21]. In a previous study of ovarian MRI, there was significant image noise on the conventional SSFSE images, even with a slice thickness of 6 mm and larger FOV [12]. However, SSFSE-DL images showed significantly improved image noise compared to the conventionally reconstructed SSFSE images, and the noise was even less than the FSE images, indicating that the image noise was adequately removed in the images reconstructed by a deep convolutional neural network-based AIR^TM^ Recon DL. This neural network model embedded in the MR image reconstruction pipeline employs a cascade of over 100 thousand unique pattern recognitions for images with noise and low resolution to reconstruct high-resolution images with high SNR [22]. Better SNR on DL reconstructed images enhanced the signal strength of the long-T2-value follicles and increased the contrast between follicles and the surrounding ovarian stroma, thus facilitating the count of the follicles. Moreover, SSFSE-DL achieved a superior contrast between pelvic fluid collection and surrounding fat tissue than FSE T2WI without fat saturation.

In this study, SSFSE-DL images showed fewer blurring artifacts when compared with the original SSFSE and FSE images using conventional reconstruction. The embedded AIR^TM^ Recon DL ahead of conventional reconstruction was one of the main reasons for our result. A conventional image-based linear filter can partially suppress ringing artifacts and noise caused by the iFT reconstruction method, while portions of the acquired k-space data are reduced for consistency with the unsampled high-frequency data, only partially removing these artifacts but broadening the point spread function, thus degrading resolution and blurring images [23]. However, AIR^TM^ Recon DL can use truncation artifacts as indicators of missing information rather than general suppression of truncation artifacts so as to make the target edge clear to the collected data [22]. In addition to the modified reconstruction pipeline, the blurring on the FSE images is mainly attributed to the inevitable motion during the relatively long acquisition time, such as the respiratory and bowel motility. In other words, imaging speed is indeed the decisive factor in reducing motion artifacts. In this study, the acquisition time for a single slice using the SSFSE sequence was less than one second; thus, both respiratory motion and bowel peristalsis were easily frozen, which significantly reduced the blurring effect related to motion. Therefore, the follicles with various sizes were well delineated on the SSFSE-DL images, making the follicle counting more easily and accurately. Similarly, the overall image quality of DL-reconstructed SSFSE for the uterus MRI was equal to or better than that of the periodically rotated overlapping parallel lines with enhanced reconstruction (PROPELLER) sequence [14]. In addition to the female pelvis, the combination of DL reconstruction and SSFSE sequence was also validated in the liver MRI depending on its superior image quality relative to that of the standard FSE sequence, despite its inferior image quality relative to that of the PROPELLER sequence [24,25].

The accurate assessment of follicle count plays an important role in the identification of PCOM for patients with suspected PCOS [26,27] and in the evaluation of ovarian reserve for infertile women [28]. Many studies have reported thresholds to identify PCOM using conventional FSE T2WI. The follicle counts of 20.5 on 0.7 × 0.7 × 6.0 mm^3^ images were recommended to identify adolescents and young adults with PCOS [29]. The maximum specificity for follicle number per section of 17 could differentiate PCOS adolescents from matched controls on images with a slice thickness of 3–4 mm [5]. The FNPO of 28 (≤9 mm) showed the best sensitivity and specificity on the images of 3.5 mm slice thickness [11]. These variable thresholds in these studies demonstrate that repeatability of the follicle count estimation dominates the superiority of the identification criteria for PCOM.

To evaluate the repeatability of follicle counting using the conventional FSE T2WI sequences, various scanning parameters were examined. In an ovarian MRI study with an acquisition voxel of about 0.7 × 0.7 × 4 mm^3^ on 1.5T, consistencies for the total follicle count were good, while the inter-observer consistency in evaluating follicles 1 to 3 mm in size was poor to moderate [9]. In another study with an acquisition voxel of about 0.7 × 0.7 × 3 mm^3^ on 3T, the intra- and inter-observer consistencies for the total follicle count were substantial, while the inter-observer consistency for follicles 1 to 6 mm in size was moderate [10]. We speculated that the mediocre performance of the conventional FSE images on the repeatability of follicle count was mainly caused by the blurring artifacts and limited spatial resolution, which hindered the discrimination of adjacent follicles, especially those of small sizes. For DL-reconstructed SSFSE in our study, the intra- and inter-observer agreements for the FNPO assessment were significantly improved and benefited from the better clarity of individual ovarian follicles compared to the widely-used FSE sequences. Moreover, the inter-observer variability comparable to the intra-observer’s variability implied that reliable follicle counting based on the SSFSE-DL images could be achieved by two different radiologists, while the inter-observer’s variability for the follicle counting using the FSE images was higher than the intra-observer’s. Therefore, SSFSE-DL images were shown to be more effective and independent of the experience of radiologists in the identification of polycystic ovaries than the conventional FSE images.

Our study had several limitations. First, only adolescent girls and adult women with clinically confirmed PCOS were included, and no control subjects were included. Moreover, it should be noted that seven of the participants had already received oral hormonal contraceptive agents for treatment prior to imaging, making it infeasible to determine thresholds for PCOM identification in our study. However, our study placed emphasis on the repeatability of the FNPO assessment that was not affected by the inclusion criterion. The identification of PCOM, especially in adolescent girls, using the SSFSE-DL sequence will be explored. Second, the follicles were not classified by size in this study. It was because the blurring artifacts may lead to the unclear delineation of the follicles, resulting in a false measurement of a single follicle or multiple follicles being considered as one. Thus, the classification of follicle size may reduce the reliability of the repeatability assessment of the follicle count, especially when there are severe artifacts. Third, although the follicle counting was performed on the axial images and with reference lines on the other two imaging planes, the respiratory movement and intestinal peristalsis may still lead to a mismatch of the images on the three different planes, potentially resulting in repeated or missed follicle counts. To mitigate the effects of slice mismatch, the incorporation of breath-hold acquisition mode and gastrointestinal motility inhibitors would be an optimal option if possible. Fourth, although the follicles were more clearly displayed on the SSFSE-DL images, the long T2 relaxation time may affect the display of other pelvic structures and the detection rate of lesions, which warrants further investigation.

In conclusion, compared with the original SSFSE images and conventional FSE images, high-resolution SSFSE images with DL reconstruction can significantly improve the repeatability and reproducibility of FNPO evaluation, benefiting from the better display of the follicles, which indicates that SSFSE-DL is a more stable and reliable method for follicle count assessment.

## Figures and Tables

**Table 1:** Image quality scores for comparison between SSFSE-DL and SSFSE and between SSFSE-DL and FSE.

Rated Feature	Observer	SSFSE-DL	SSFSE	FSE	SSFSE-DL vs. SSFSE	SSFSE-DL vs. FSE
Blurring artifacts	1	2.600 ± 0.598	2.200 ± 0.834	1.950 ± 0.826	*p* = 0.0117	*p* = 0.0015
	2	2.550 ± 0.510	2.050 ± 0.759	1.900 ± 0.788	*p* = 0.0051	*p* = 0.0022
Inter-observer agreement		0.720 (0.463–0.976)	0.591 (0.338–0.843)	0.474 (0.165–0.783)		
Subjective noise	1	2.750 ± 0.550	1.500 ± 0.513	2.450 ± 0.605	*p* = 0.0001	*p* = 0.0277
	2	2.650 ± 0.587	1.700 ± 0.571	2.250 ± 0.550	*p* = 0.0001	*p* = 0.0051
Inter-observer agreement		0.789 (0.498–1.000)	0.636 (0.352–0.921)	0.664 (0.366–0.961)		
Clarity of the follicles	1	4.150 ± 0.745	3.700 ± 0.657	3.150 ± 0.875	*p* = 0.0164	*p* = 0.0003
	2	4.400 ± 0.681	3.800 ± 0.696	3.200 ± 1.005	*p* = 0.0051	*p* = 0.0007
Inter-observer agreement		0.671 (0.423–0.919)	0.565 (0.245–0.885)	0.536 (0.260–0.812)		

Note. Data are presented as mean ± SD. Comparisons of the subjective scoring were performed using the Wilcoxon signed-rank test. Cohen’s weighted kappa (95% confidence interval) values are shown for inter-observer agreement.

**Table 2:** The descriptive statistics for follicle number per ovary.

Sequence	Observer	Session	Minimum	Maximum	Mean	SD
SSFSE-DL	1	1	12	38	24.7	5.4
	2	2	14	35	24.4	5.0
	1	3	14	36	24.3	4.8
SSFSE	1	1	12	32	22.1	5.0
	2	2	14	31	21.6	4.8
	1	3	13	32	20.9	4.2
FSE	1	1	13	32	20.8	4.1
	2	2	12	34	21.6	4.7
	1	3	11	30	20.6	5.0

**Table 3:** The intra-observer and inter-observer agreements for FNPO assessment based on each image type.

Sequence	Intra-Observer ICC (95% CI)	Inter-Observer ICC (95% CI)
SSFSE-DL	0.930 (0.878–0.962)	0.914 (0.843–0.953)
SSFSE	0.825 (0.693–0.903)	0.798 (0.650–0.888)
FSE	0.839 (0.716–0.912)	0.669 (0.454–0.810)

Note. Comparisons of the intra-observer and inter-observer agreements were performed using the intra-class correlation coefficients. Intra-observer and inter-observer ICC were calculated based on the results of FNPO evaluation, respectively, of the first and second session by observer 1 and of the first session by viewer 1 and reviewer 2.

**Table 4:** Comparison of the absolute values of intra-observer and inter-observer differences.

Parameter	SSFSE-DL	SSFSE	FSE	SSFSE-DL vs. SSFSE	SSFSE-DL vs. FSE
Intra-observer difference	1.475 ± 1.062	2.300 ± 1.772	2.325 ± 1.163	*p* = 0.0185	*p* = 0.0001
Inter-observer difference	1.900 ± 0.955 ^NS^	2.825 ± 1.412 ^NS^	3.325 ± 1.655 *	*p* = 0.0004	*p* < 0.0001

Note. Comparisons were performed using a paired *t* test. ^NS^ *p* > 0.05 for comparison between intra-observer and inter-observer differences. * *p* < 0.05 for comparison between intra-observer and inter-observer differences.

## Data Availability

The datasets analyzed during the current study are available from the corresponding author on reasonable request.
